# Multiparticulate System for Colon Targeted Delivery of Ondansetron

**DOI:** 10.4103/0250-474X.62237

**Published:** 2010

**Authors:** S. Jose, K. Dhanya, T. A. Cinu, N. A. Aleykutty

**Affiliations:** Department of Pharmaceutical Sciences, Cheruvandoor campus, Mahatma Gandhi University, Ettumanoor, Kottayam-686 631, India

**Keywords:** Chitosan microspheres, colon targeted drug delivery, multiparticulate systems, ondansetron

## Abstract

Targeted delivery of drugs to colon has the potential for local treatment of a variety of colonic diseases. The main objective of the study was to develop a multiparticulate system containing chitosan microspheres for the colon targeted delivery of ondansetron for the treatment of irritable bowel syndrome. This work combines pH-dependent solubility of eudragit S-100 polymers and microbial degradability of chitosan polymers. Chitosan microspheres containing ondansetron were prepared by emulsion cross linking method. The effect of process variables like chitosan concentration, drug-polymer ratio, emulsifier concentration and stirring speed were studied on particle size and entrapment efficiency of chitosan microspheres. *In vitro* drug release studies in simulated gastro intestinal fluids showed a burst drug release pattern in the initial hour necessitating microencapsulation around the chitosan microspheres. The optimized formulation was then subjected to microencapsulation with eudragit S-100 by solvent evaporation technique. The effect of different coat/core ratio on particle size, drug entrapment efficiency and *in vitro* drug release were studied. Formulation which contain 1:10 core/coat ratio released lesser amount of drug in the upper gastro intestinal conditions and so selected as best formulation and then subjected to *in vitro* drug release studies in presence of rat ceacal contents to assess biodegradability of chitosan microspheres in colon. In order to study the drug release mechanism *in vitro* drug release data was fitted into various kinetic models. Analysis of regression values suggested that the possible drug release mechanism was Peppas model.

Oral colon-targeted drug-delivery systems have recently gained importance for delivering a variety of therapeutic agents for both local and systemic administration. Targeted delivery of drugs to colon has the potential for local treatment of a variety of colonic diseases such as irritable bowel syndrome (IBS), colorectal cancer and inflammatory bowel diseases (IBD) that includes both ulcerative colitis and Crohn's disease. Apart from this local treatment, colon is used for the systemic absorption of proteins and peptides because of the less hydrolytic hostile environment in comparison with stomach and small intestine as well as the existence of specific transporters. Also colon is a good site for those drugs where a delay in drug absorption is required from therapeutic point of view e.g. in case of nocturnal asthma, arthritis, cardiac arrhythmias which are effected by circadian biorhythms[1]. Additionally, the colon is a highly responsive site for the absorption of poorly absorbable drugs[2]. By this colon targeted drug delivery it is also possible to prevent the side effects of drugs on healthy tissues and enhancement of drug uptake by targeted cells. The dose can be reduced thus decreases the toxicity and cost if the active drug is costly.

Multiparticulate approaches include formulations in the form of pellets, granules, beads, microparticles and nanoparticles. Recently, much emphasis is being laid on the development of multiparticulate dosage forms in comparison to single unit systems because of their potential benefits like increased bioavailability, reduced risk of systemic toxicity, reduced risk of local irritation, predictable gastric emptying and retained in the ascending colon for a relatively long period of time. Because of their smaller particle size as compared to single unit dosage forms these systems are capable of passing through the GI tract easily, leading to less inter- and intra subject variability. Moreover, multiparticulate systems tend to be more uniformly dispersed in the GI tract and also ensure more uniform drug absorption[3]. Single unit colon targeted drug delivery system may suffer from the disadvantage of unintentional disintegration of the formulation due to manufacturing deficiency or unusual gastric physiology that may lead to drastically compromised systemic drug bioavailability or loss of local therapeutic action in the colon.

5-HT3 receptors are thought to be involved in the regulation of gastrointestinal motor function through its action on nerve receptors within the enteric nervous system and in the modulation of visceral sensory function. Ondansetron, a 5-HT3 receptor antagonist appears to reduce visceral sensitivity and have inhibitory effects on motor activity in the distal intestine and may have a wider application in the prophylaxis and treatment of nausea and vomiting. It has been demonstrated that ondansetron is well absorbed in the intestinal segments including the upper small intestine, the colon and the rectum. In a randomized, double blind cross-over placebo controlled study of ondansetron 16 mg tid, patients with IBS experienced significantly fewer daily episodes of pain while on ondansetron. Ondansetron caused firmer bowel movements[4]. Targeting of ondansetron to the colon may provide adequate treatment for IBS and allow a reduction in dosage and possible systemic side effects[5].

Successful targeted delivery of drugs to the colon via the gastrointestinal tract requires the protection of a drug from degradation, release and/or absorption in stomach and small intestine and then ensures abrupt or controlled release in the proximal colon[3]. This might be achieved by the use of specially designed drug delivery system (DDS) that can protect the drug during its transfer to colon. In this work, a multiparticulate system of ondansetron for the treatment of irritable bowel syndrome was developed by utilizing the pH-dependent solubility of Eudragit S-100 polymers and microbial degradability of chitosan polymers. Ondansetron-loaded chitosan microspheres were prepared, which is then microencapsulated with eudragit S-100 polymer. This polymer shows the solubility at or above pH 7. So that the microencapsulated system become soluble at this pH and releases the chitosan microspheres. The microbial degradation of the chitosan microspheres in the colon releases the drug in the targeted site.

## MATERIALS AND METHODS

Ondansetron (Wallace Pharmaceuticals Ltd, Goa, India), chitosan (CIFT, Cochin, India), Eudragit S 100 (Matrix Laboratories Ltd. Hyderabad, India) were obtained as gift samples. Glacial acetic acid and Span 80 were purchased from Central Drug House, New Delhi, India. Heavy and Light liquid paraffin, methanol, petroleum ether and acetone were procured from Nice Chemicals Cochin, India. Wistar rats were purchased from Veterinary College, Mannuthy, Kerala, India and were housed in the animal house of Department of Pharmaceutical Sciences, Cheruvandoor, Kerala, India.

### Preparation of chitosan microspheres:

The chitosan microspheres were prepared by emulsion cross-linking method. Chitosan solution was prepared in aqueous glacial acetic acid by over night stirring in a magnetic stirrer. The drug was dispersed in this solution and mixed well. Resultant mixture was then injected through a syringe into 20 ml of oil phase containing Span 80 and stirring was performed by mechanical stirrer at 1500 rpm to form w/o emulsion. Oil phase was a mixture of heavy and light liquid paraffins. After 30 min homogenization period toluene-saturated gluteraldehyde was added to it stage by stage. It was then left for stabilization and cross-linking for a period of 7 h. Microspheres thus obtained were centrifuged at 4000 rpm and the sediment was then washed with petroleum ether and acetone and then dried in a hot air oven at 50°.

### Analysis of particle size, shape and surface morphology:

The particle size of all the microspheres were evaluated using optical microscope fitted with a calibrated eyepiece micrometer. The average particle size was determined by the Edmondson's Eqn[6]. The shape and surface morphology of the chitosan microspheres was studied using a Jeol JSM-6390 scanning electron microscope (Jeol, Tokyo, Japan).

### Entrapment efficiency[7,8]:

Microspheres were accurately weighed and triturated with methanol and kept over night for extraction of drug for the determination of entrapment efficiency. After filtration and appropriate dilution with methanol the absorbance was measured with UV/Vis spectrophotometer (Spectroscan 2600, Chemito, India) at 302 nm.

### *In vitro* drug release studies in simulated gastro intestinal fluids[9,10]:

**Scheme 1:**
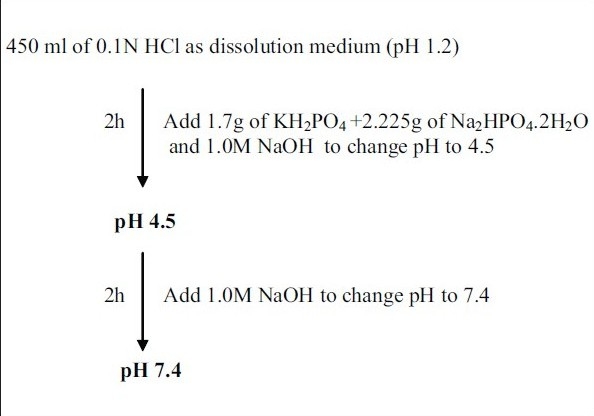
*In vitro* drug release studies in simulated gastro intestinal fluids USP rotating paddle dissolution apparatus at 100 rpm and at 37±0.5°

Chitosan microspheres were evaluated for the *in vitro* drug release in simulated gastrointestinal fluids. An accurately weighed amount of microspheres was added to 450 ml of dissolution medium and the release of ondansetron from the microspheres were investigated using the USP rotating paddle dissolution apparatus at 100 rpm and 37±0.5°. The simulation of gastrointestinal transit conditions was achieved by altering the pH of the dissolution medium at various time intervals. The pH of the dissolution medium was kept at 1.2 for 2 h with 0.1 N HCl. Then, the pH of the medium adjusted to 4.5 by adding 1.7 g of KH_2_PO_4_ and 2.225 g of Na_2_HPO_4_.2H_2_O and sufficient quantity of 1.0 M NaOH. The release rate study was continued for another 2 h. The pH of the dissolution medium was then adjusted to 7.4 by adding 1.0 M NaOH. The flow chart for the same is given in scheme 1. Two milliliter of samples were withdrawn from the dissolution medium at various time intervals for 24 h and replaced with fresh dissolution medium. The samples were then subjected to UV analysis as described previously. All dissolution studies were performed in triplicate. The effects of drug-polymer ratio on *in vitro* drug release of chitosan microspheres were evaluated.

### Microencapsulation of chitosan microspheres:

The ondansetron loaded chitosan microspheres were microencapsulated by emulsion–solvent evaporation technique. The chitosan microspheres were suspended in eudragit S-100 ethanol solution (10% w/v) and then emulsified into 40 ml of light liquid paraffin containing span 80. The emulsification process was carried out for 3 h at 1000 rpm with mechanical stirrer. The eudragit S-100 coated microspheres were collected and rinsed with petroleum ether and dried in a hot air oven at 50°.

After the microencapsulation the eudragit S-100 microencapsulated chitosan microspheres were evaluated for the particle size, shape, surface morphology, percentage yield, entrapment efficiency and *in vitro* drug release in simulated gastro intestinal fluids. Additionally *in vitro* drug release studies in presence of rat caecal contents were carried out to assess the biodegradability of chitosan by colonic bacteria.

### *In vitro* drug release studies in the presence of rat caecal contents[9,10]:

Before starting the experiments on animals, the experimental protocol was cleared by the Institutional Animal Ethical Committee (IAEC/MGU/CHE-M.pharm/001/2008 dated 04/03/2008). Four Wistar rats of body weight (150-200 g) with no prior drug treatment, were used for all the present *ex vivo* studies and maintained on normal diet, and administered 1 ml of 2% dispersion of chitosan in water, and this treatment was continued for 7 days in order to induce the enzymes that specifically act on the chitosan. Thirty minutes before starting the study, each rat was sacrificed and the abdomen was opened. The caecum was traced, legated at both ends, dissected, and immediately transferred into phosphate buffered saline (PBS) pH 6.8, which was previously bubbled with CO_2_. The caecal bag was opened; the contents were weighed, homogenized, and then suspended in simulated intestinal fluid of pH 7.4 to give the desired concentration of 2% caecal content, which was used as simulated colonic fluid. The experiment was carried out with a continuous supply of carbon dioxide into the dissolution media. Drug release studies for first 4 h were performed as described under section describing *In vitro* drug release studies in simulated gastro intestinal fluids. After 4 h the release studies were carried out in simulated intestinal fluid containing rat caecal content. Aliquots of samples were withdrawn periodically and replaced with fresh buffer bubbled with carbon dioxide. The samples were filtered through a Whatman filter paper, and drug content was determined spectrophotometrically.

## RESULTS AND DISCUSSIONS

**Fig. 1:**
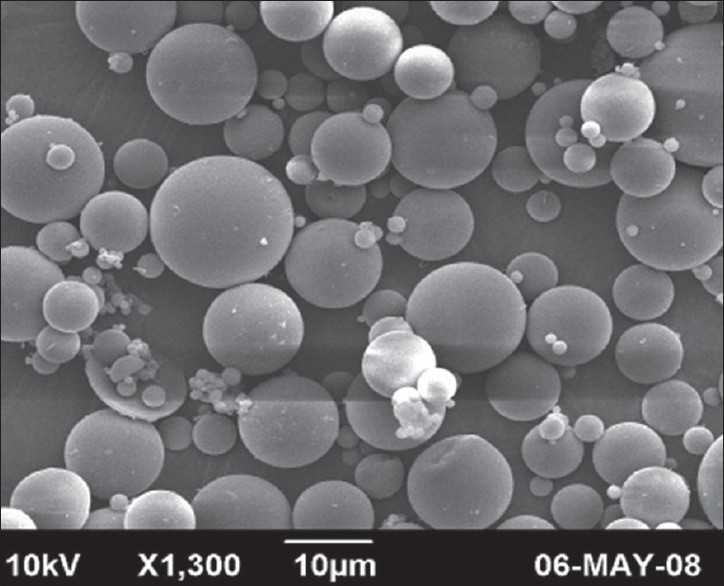
SEM photomicrograph of Chitosan microspheres Scanning electron micrograph of a group of Chitosan microspheres prepared.

The chitosan microspheres were successfully prepared by emulsion cross linking method. Microscopic analysis was performed to determine the average particle size of chitosan microspheres. The average particle size of different chitosan microsphere formulations was found to be in the range of 6.16 μm -12.56 μm (Table 1). The effects of process variables like chitosan concentration, drug-polymer ratio, stirring speed and emulsifier concentration on average particle size of chitosan microspheres were studied. The chitosan solution was found to be too viscous to pass through the needle at 3% polymer concentration (formulation C3). Hence it was avoided in the initial step itself. As the chitosan concentration increases from 1-2%, the average particle size of chitosan microspheres increases from 7.12 μm to 9.52 μm. The mean diameter of the microspheres was increased from 6.8 μm to 9.52 μm with increasing the drug- polymer ratio from 1:2 to 1:10. This increase in particle size can be attributed to an increase in viscosity with increase in polymer concentration, which resulted in larger emulsion droplets and finally in greater microsphere size. The particle size was found to be decreased from 12.56 μm to 5.52 μm with increasing stirring speed from 1000 to 2000 rpm. The result may be due to high stirring rate, which provides the shearing force, needed to reduce size of emulsion droplet. The average particle size of microspheres was found to be decreased from 11.12 μm to 6.16 μm with increasing emulsifier concentration. The decrease in particle size with increase in emulsifier concentration may be due to its surfactant property. Emulsifier has the ability to stabilize the interface between two phases. As the emulsifier concentration increases, it allows to stabilize a greater surface area, thus leading to smaller particle size (Table 1). The SEM studies of chitosan microspheres showed that they were spherical in shape and with a smooth surface. The SEM photographs of chitosan microspheres are shown in (fig. 1).

**TABLE 1:** AVERAGE PARTICLE SIZE AND ENTRAPMENT EFFICIENCY OF DIFFERENT CHITOSAN MICROSPHERE FORMULATIONS

Parameters	Process variables	Formulation code	Average particle size (μm)	Entrapment efficiency (%)*
Chitosan concentration	1% W/V	C1	7.12	67.33±0.81
	2% W/V	C2	9.52	72.80±0.83^a^
Drug-Polymer ratio	1:2	D1	6.80	60.17±0.25
	1:4	D2	7.28	62.43±0.52^a^
	1:6	D3	8.40	65.89±0.92^a^
	1:8	D4	8.96	70.34±0.68^a^
	1:10	D5	9.52	72.80±0.83^a^
Stirring speed	1000	S1	12.56	69.95±1.04
	1500	S2	9.52	72.80±0.83^a^
	2000	S3	5.52	72.11±0.69^ns^
Emulsifier concentration	0.5% W/V	E1	11.12	71.95±0.74
	1% W/V	E2	9.52	72.80±0.83^ns^
	1.5% W/V	E3	6.16	72.03±0.48^ns^

* One way analysis of variance test (Dunnett's test) was conducted on all data. Data shown are mean of three readings standard deviation.

a p<0.05

ns - non significant

The drug entrapment efficiency of different formulations was found to be between 60-73%. The effects of 1% and 2% chitosan concentrations were studied and the entrapment efficiency was higher (72.80±0.83%) for formulation C2 which contain 2% chitosan concentration. This showed that high drug entrapment was observed at higher chitosan concentration. The effect of drug-polymer ratios (1:2, 1:4, 1:6, 1:8 and 1:10) on entrapment efficiency of chitosan microspheres were studied and high entrapment efficiency was observed for D5 formulation that contain higher drug polymer ratio. The effect of stirring speed on entrapment efficiency of chitosan microspheres showed that optimum speed should be 1500 rpm. Evaluation of entrapment efficiency of formulations (E1- E3) prepared by varying emulsifier concentrations revealed that emulsifier concentrations do not have any significant effect on drug loading (Table 1).

**Fig. 2:**
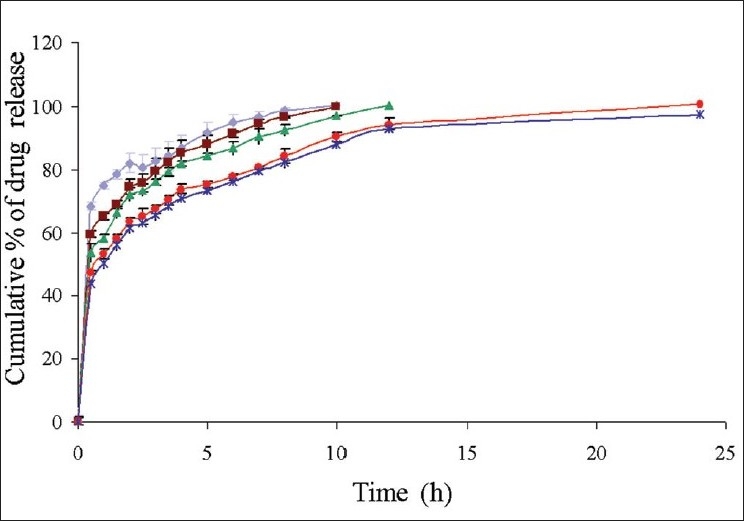
Cumulative *in vitro* drug release of ondansetron from formulations 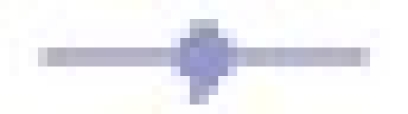
 D1drug:polymer ratio 1:2, 
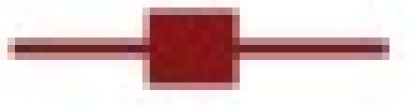
 D2 drug: polymer ratio 1:4, 
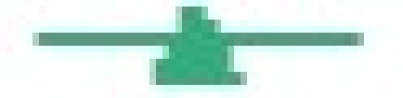
 D3 drug:polymer ratio 1:6, 
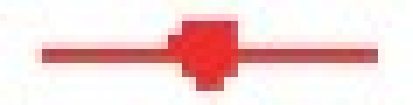
 D4 drug:polymer ratio 1:8, 
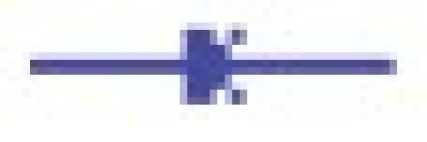
 D5 drug:polymer ratio 1:10.

The *in vitro* drug release studies of D1-D5 formulations in simulated gastro intestinal fluids showed a burst release pattern in the initial hour (fig. 2). A high burst release of 68.40±1.86% was observed from the formulation D1, which contain 1:2 drug polymer ratio; whereas a less burst release of 43.71±1.28 was observed from D5. These results indicated that formulation with lesser drug–polymer ratio shows higher drug release. Within 4 h 70-90% of drug was released from the formulations D1-D5. This type of high drug release in stomach and small intestine is not satisfactory for a formulation, which is supposed to release its contents in the colon. The burst release may be due to solubility of chitosan in the acidic pH. In order to prevent the drug release in stomach and small intestine these chitosan microspheres were encapsulated with Eudragit S-100, which shows solubility at a pH ≥7. Since D5 formulation showed high drug loading and drug release pattern, it is selected for microencapsulation process.

**Fig. 3:**
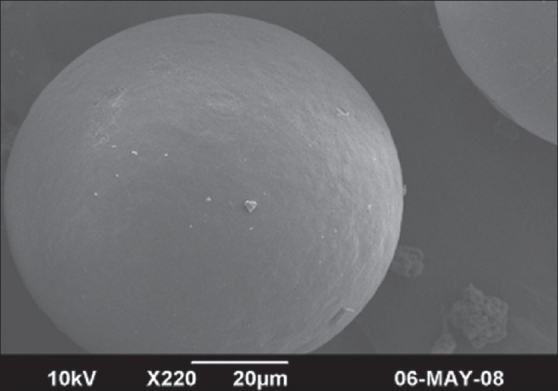
SEM photomicrograph of chitosan microspheres Scanning electron micrograph of a single Eudragit S- 100 microencapsulated chitosan microsphere

Chitosan microspheres (D5) were microencapsulated with eudragit S-100 to achieve colon targeted delivery of ondansetron. The effect of core-coat ratio on eudragit S-100 microencapsulated chitosan microspheres was studied and found that the particle size was increased from 121 μm to 155 μm with increasing the core-coat ratio from 1:6 to 1:12. This increase in particle size may be due to corresponding increase in the polymer concentration that results in larger emulsion droplets (Table 2). The SEM study showed that Eudragit S-100 microencapsulated chitosan microspheres exhibited smooth surface and spherical shape (fig. 3).

**TABLE 2:** AVERAGE PARTICLE SIZE AND ENTRAPMENT EFFICIENCY OF MICROENCAPSULATED FORMULATIONS

Coating polymer	Core/Coat ratio	Formulation code	Average particle size (μm)	Entrapment efficiency (%)*
Eudragit S-100	1:6	EC1	121	91.08±1.90
	1:8	EC2	136.5	93.19±1.63
	1:10	EC3	148	94.68±2.23
	1:12	EC4	155	94.36±1.94

* Data shown are mean of three readings±standard deviation

The entrapment efficiency of microencapsulated formulations varied between 90-95% with increasing core-coat ratio (Table 2). The high entrapment efficiency was found in formulation EC3 containing 1:10 core-coat ratio. When the core-coat ratio was increased into 1:12 a slight fall in drug entrapment efficiency was observed.

**Fig. 4:**
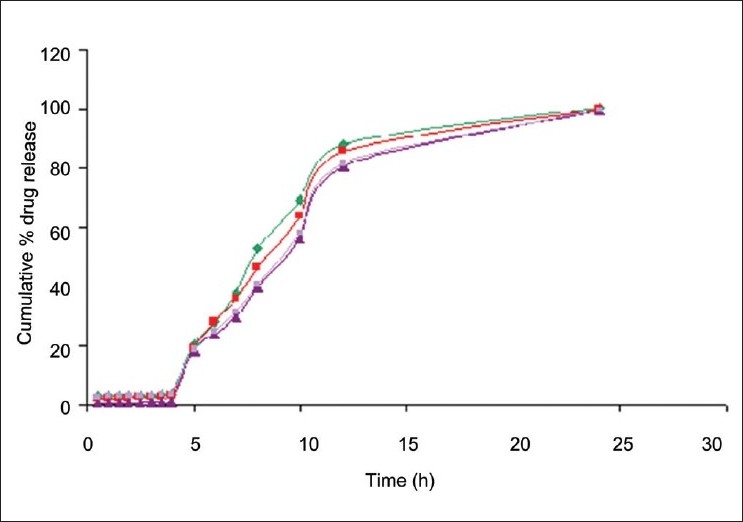
Cumulative *in vitro* drug release of ondansetron from microencapsulated chitosan microspheres. 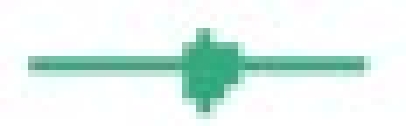
 EC1 core:coat ratio 1:6, 
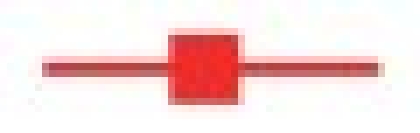
 EC2 core:coat ratio 1:8, 
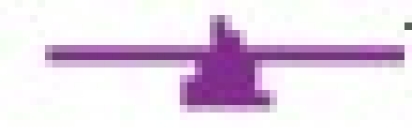
 EC3 core:coat ratio 1:10, 
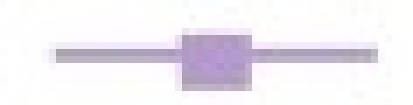
 EC4 core:cat ratio 1:12

The *in vitro* drug release studies of various eudragit coated chitosan microspheres were performed in simulated gastro intestinal fluids and shown in (fig. 4). The effects of core-coat ratio on in vitro drug release were studied. The drug release studies indicated that eudragit S-100 coating around the chitosan microspheres offers a high degree of protection from premature drug release in the stomach and small intestine. Results showed that 8.09±0.89 to 12.06±1.21% of drug was released within initial 4 h from the formulations EC2 and EC1, respectively. The drug release rate was increased after 4 h, because at that time formulations were exposed to pH 7.4, which is above the solubility of the eudragit S-100 polymers. EC3 formulation containing 1:10 core/coat ratio releases only 3.64±0.34% of ondansetron within 4 h. A similar drug release pattern was observed for formulation containing 1:12 ratio. This indicates that both EC3 and EC4 formulations releases less amount of drug in stomach and small intestine when compared to other formulations. Since EC3 contain 1:10 ratio and was also able to maintain drug release pattern similar to EC4, EC3 formulation was selected as the best formulation and then subjected to *in vitro* drug release studies in presence of rat ceacal contents.

**Fig. 5:**
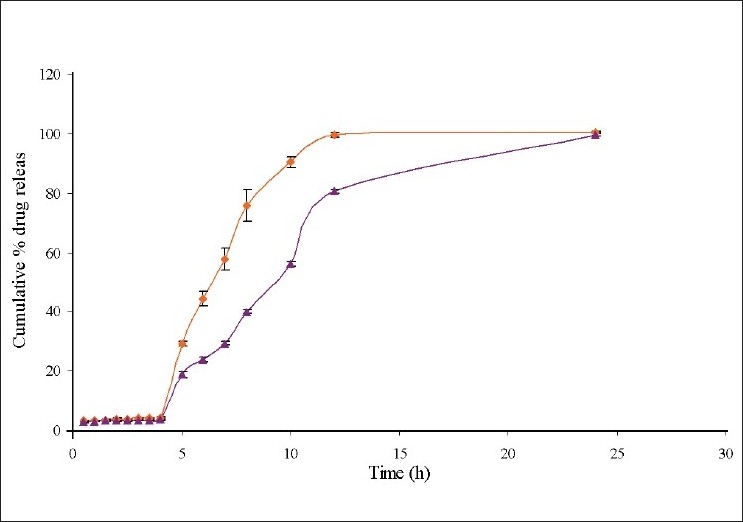
Comparative *In Vitro* drug release profile of EC3 with and without rat caecal contents 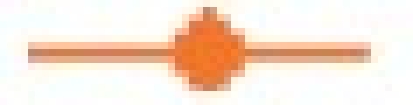
 EC3-with rat cecal contents core: coat ratio 1:10, EC3-without rat cecal contents 
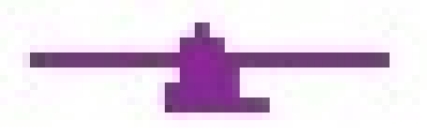
 core: coat ratio 1:10

Biodegradability of chitosan microspheres to colonic bacteria was evaluated by performing *in vitro* drug release study of formulation EC3 in the presence of rat caecal contents and the drug release data obtained with and with out rat caecal contents were compared. This revealed that the drug release was improved when it is carried out in the presence of rat caecal contents. Around 99.52±0.89% of drug was released within 12 h and the rapid release of drug was due to action of colonic microflora (fig. 5).

Since EC3 was the best formulation drug release mechanisms were determined by fitting *in vitro* drug release data to various the kinetic models. The curvilinear nature of the cumulative percentage drug released vs time plot suggests that EC3 formulation does not follow zero order drug release kinetics. This was again confirmed by poor correlation coefficient value when compared to other kinetic models. By comparing correlation coefficient values for first order, Higuchi model and Peppas model EC3 formulation gave good fit to the Korsmeyer- Peppas model. Since the diffusion exponent (n) value was greater than 1, the drug release follows super case II transport. This model is used to analyze the release of pharmaceutical dosage forms when the release mechanism is not well known or when more than one type of release phenomena was involved.
